# Cluster-ID-Based Throughput Improvement in Cognitive Radio Networks for 5G and Beyond-5G IoT Applications

**DOI:** 10.3390/mi13091414

**Published:** 2022-08-28

**Authors:** Stalin Allwin Devaraj, Kambatty Bojan Gurumoorthy, Pradeep Kumar, Wilson Stalin Jacob, Prince Jenifer Darling Rosita, Tanweer Ali

**Affiliations:** 1 Department of Electronics and Communication Engineering, Francis Xavier Engineering College, Tirunelveli 627003, India; 2 Department of Electronics and Communication Engineering, KPR Institute of Engineering and Technology, Coimbatore 641407, India; 3 Discipline of Electrical, Electronic and Computer Engineering, University of KwaZulu-Natal, Durban 4041, South Africa; 4 Engineering Department—Electrical Engineering, Botho University, Gaborone 501564, Botswana; 5 Electrical Engineering Department, New Era College, Gaborone 501564, Botswana; 6 Department of Electronics and Communication Engineering, Manipal Institute of Technology, Manipal Academy of Higher Education, Manipal 576104, India

**Keywords:** cluster-based cooperative spectrum sensing, achievable throughput, greedy heuristic algorithm, cognitive radio network, 5G and beyond-5G IoT applications

## Abstract

Cognitive radio (CR), which is a common form of wireless communication, consists of a transceiver that is intelligently capable of detecting which communication channels are available to use and which are not. After this detection process, the transceiver avoids the occupied channels while simultaneously moving into the empty ones. Hence, spectrum shortage and underutilization are key problems that the CR can be proposed to address. In order to obtain a good idea of the spectrum usage in the area where the CRs are located, cooperative spectrum sensing (CSS) can be used. Hence, the primary objective of this research work is to increase the realizable throughput via the cluster-based cooperative spectrum sensing (CBCSS) algorithm. The proposed scheme is anticipated to acquire advanced achievable throughput for 5G and beyond-5G Internet of Things (IoT) applications. Performance parameters, such as achievable throughput, the average number of clusters and energy, have been analyzed for the proposed CBCSS and compared with optimal algorithms.

## 1. Introduction

Cognitive radio (CR) is a relatively new long-haul radio innovation. After the software-defined radio (SDR), which is, to a greater degree, gradually becoming a reality, CR will be the more successful radio correspondence framework to be created [1,2,3,4,5,6,7,8,9,10]. The principal focal point of this research work is the range designation issue, i.e., to accurately choose the Secondary Users (SUs) to detect and use the Primary User (PU) channels for a wide variety of situations [11,12,13,14,15,16,17,18,19,20,21,22].

Since the CR is being used to provide a strategy for utilizing the range more effectively, range detection is a significant issue in 5G and beyond-5G IoT applications, and the frequency band used is narrow band LTE (NB-LTE) [23,24,25]. The general framework is to work adequately and to provide the necessary enhancement in range detection. The CR range detection framework must have the option to successfully distinguish the additional transmissions, recognize them and enlighten the central processing unit of the CR such that the crucial move can be made.

This work addresses the following:

- Methods of increasing the throughput of SUs.
- A discussion of the spectrum utilization problem.
- The proposal of cluster-based cooperative spectrum sensing (CBCSS) to perform cooperative spectrum sensing in 5G and beyond-5G IoT applications.

To extract the minimum-sensing users, a closed-form expression is logically impractical, and a number of security issues need to be considered. A higher dimensions vector is not considered here since it increases the computational cost and makes the algorithm undesirable.

## 2. Literature Survey

The extraction of the number of minimum-sensing users in a closed-form expression is analytically unfeasible. Therefore, the analytical formulation of the amount of saved energy in each scenario must be considered. In addition, a greater number of security issues are considered. The higher dimensions vector is not considered, and if considered, it increases the computational cost, which makes the algorithm undesirable. There are also practical protocol issues of synchronization and the estimation and tracking of the traffic parameters. In addition to spectrum sensing to improve spectrum utilization, a CR in a cognitive radio network (CRN) can sense available networks and communication systems around it. The CRNs are composed of various kinds of communication systems and networks and can be viewed as a sort of heterogeneous network. Dusit Niyato et al. recommended a Bertrand game model to analyze the impacts of system parameters such as spectrum substitutability and channel [7]. Nasif et al. offered an algorithm for opportunistic spectrum sharing with multiple co-channel primary transmitters. The authors presented a distributed collaborative algorithm for cognitive radios [14]. Quan et al. proposed a soft computing-based algorithm that requires full consideration of the noise level of all secondary users. The throughput performance of SUs in cognitive radio networks has been analyzed [17]. Yue Wang et al. proposed an anti-jamming problem in the presence of a smart jammer. This smart jammer learns the transmission power of the user and adjusts its transmission power to maximize the damaging effect that is being analyzed [4,21]. Mohammad Rashid et al. developed a framework to learn the QoS performance measures using a queuing analysis in the data link layer for infrastructure-based cognitive radio users in the opportunistic spectrum access [12]. Song et al. considered the interference temperature restriction and opportunistic spectrum allocation and suggested a suitable framework for the joint spectrum allocation and power control to make use of the utilized and underutilized licensed electromagnetic spectrum [19]. 

Bin Wang et al. proposed an approach to enhance the performance of unlicensed users by utilizing the licensed user spectrum in cognitive radio networks [3]. Minh-Viet Nguyen et al. investigate the problem of resource allocation spectrum sensing methods, frequency selectivity, spectrum allocation and frequency bands in cognitive radio relay networks [11]. Azarfar et al. proposed an auction approach along with an anticipated double spectrum to augment the spectrum utilization in a CR network [2]. Li et al. recommended a new cooperative spectrum sensing framework that would effectively combine spectrum sensing and spectrum sharing [10]. Ruby et al. introduced a new hard two-bit overhead for each individual user. Consequently, a balanced trade-off between the detection performance and complexity was realized [18]. Dongyue Xue et al. proposed a scheduling algorithm to reduce the spectrum sensing time for wireless mesh applications [6]. Changpeng Ji et al. proposed a cross-layer cluster-based spectrum sensing algorithm to achieve better noise density and slot length [5]. Li Yu et al. proposed a framework in order to increase the performance of a CR network by using the cluster-based cooperative spectrum sensing technique [9]. Parzy et al. employed a distributed resource allocation mechanism for CR networks based on a novel competition methodology, which syndicates the benefits of node competition and cooperation [16]. Nadine Abbas et al. presented the spectrum availability and scarcity in the radio spectrum for cognitive radio networks [13]. Tsakmalis et al. extended an algorithm to increase the cognitive radio network throughput and reduce the primary user interference through scheduling techniques [20]. Zhu et al. considered the resource allocation issue in an OFDMA-based cognitive radio network and increased the coverage area of the antenna using several beam forming techniques to support the secondary user in using the licensed user spectrum [22]. Orumwense et al. proposed a cooperative technique to address the spectrum sensing issues of secondary users. The primary user channel length, spectrum sensing time and slot length were all measured for the spectrum allocation decision for secondary users [15]. Alberti et al. concluded that the problem of sensor applications in cognitive radio networks is that the parameters considered provide a short network lifetime and poor beamforming characteristics [1]. Heejung et al. presented a survey on the next generation of Internet of Things (IoT) networks, showing an increase in delay, network lifetime, packet delivery ratio and throughput [8].

In this paper, the heterogeneities in both PU channels and SUs are investigated. The PU channel is characterized by channel idle probability and channel capacity, while the SU is depicted by the energy detection threshold, received SNR and geographical location.

## 3. Cluster-Based Cooperative Spectrum Sensing (CBCSS)

### System Model

**Figure 1:**
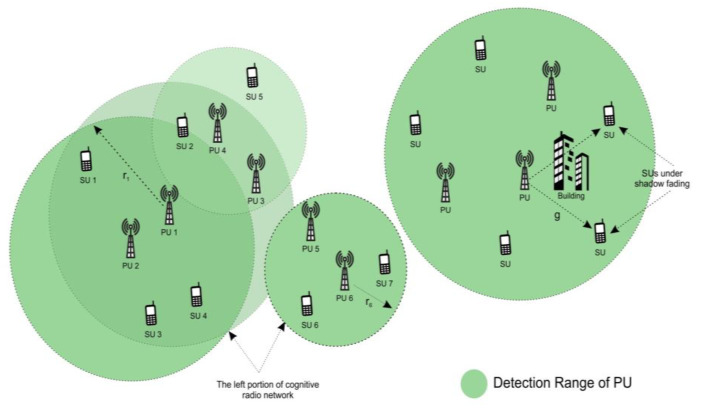
System model for CBCSS.

Consider a CRN with N SUs and M PU channels. Each channel is exclusively used by the PU. However, the PU is idle, and the SU can opportunistically utilize the channel when it is available through spectrum sensing. Let M be the set of such PU channels and N denote the set of SUs. Figure 1 demonstrates that the channel heterogeneity-spectrum availability varies across the SUs. The SUs that are located far from the PU will only report noise when the detection range of the PUs only covers part of the system. Hence, the CRN is partitioned into clusters so that the SUs in each cluster are within the detection range of the same set of PU channels. 

The vacant portion of the spectrum can only be allotted by a CR user. We use a sampling frequency ${\;f}_{s\;}$ to sample the frequency.

(1) $$H_{j}^{1}:{\;y}_{i,j}\left(k\right)={\;s}_{i,j}\left(k\right)+{\;u}_{i,j}\left(k\right)\;i=1,2,\ldots \;\ldots ,N\;$$

(2) $$H_{j}^{0}:{\;y}_{i,j}\left(k\right)={\;u}_{i,j}\left(k\right)\;j=1,2,\ldots \;\ldots ,N\;$$

The false alarm probability $P_{f,\left(i,j\right)}\;$ is defined as the probability of the SU j under $H_{j}^{0}$, which is given by

(3) $$P_{f,\left(i,j\right)}=Q\;((\frac{\in_{i}}{\sigma_{u_{i,j}}^{2}}\;-\;1)\;\sqrt{f_{s}\;T}\;)$$

The detection probability $\;P_{d,\left(i,j\right)}$ is defined as

(4) $$P_{d,\left(i,j\right)}=Q\;((\frac{\in_{i}}{\sigma_{u_{i,j}}^{2}}\;-\;1\;-\;\gamma_{i,j})\;\sqrt{\frac{f_{s}T\;}{2_{\gamma i,j}+1}})$$

In order to provide sufficient protection to the Pus, it is required to keep the detection probability above a given threshold $Q_{\mathrm{th}}$, that is, $Q_{d,j}\geq Q_{\mathrm{th}}$. Hence,

(5) $$\overset{m}{\underset{i=1}{\prod }}(1-P_{d,\left(i,j\right)})\geq {\;Q}_{\mathrm{th}\;}$$

The SUs and PU channel’s allocation matrices are ${\left[X_{s}\right]}_{N\;X\;K\;}\;\mathrm{and}\;{\left[X_{c}\right]}_{M\;X\;K\;}$. The elements $x_{s,i}^{k}$ and $x_{c,j}^{k}$ can be defined as:

(6) $$x_{s,i}^{k}\{\begin{array}{c} 1\;if\;SU\;is\;with\;cluster\;\\ r\\ 0\;otherwise \end{array}$$

(7) $$x_{c,j}^{k}\{\begin{array}{c} 1\;if\;CH_{j}\;is\;with\;cluster\;\\ r\\ 0\;otherwise \end{array}$$

Consider the following two vectors:

$S_{k}$ represents the set of SUs in cluster k

(8) $$S_{k}=\{i|x_{s,i}^{k}=\;1,\;\forall \;i\;\in \;N\}$$

$B_{k}\;$ denotes the set of PU channels sensed and utilized by the SUs in cluster k

(9) $${\;B}_{k}=\{j|x_{c,j}^{k}=\;1,\;\forall \;j\;\in \;M\}\;$$

Thus, the total throughput is given by

(10) $$R_{k}(S_{k},B_{k})=\;\sum_{j\text{£}\mathrm{Bk}}\frac{T-\tau }{T}P\;(H_{j})\;C_{j}(1-Q_{f,j}^{k}(S_{k},\;B_{k}))\;$$

where P($H_{j}$) is the idle probability for channel j, $C_{j\;}$ is the transmission capacity for channel j, and

(11) $$Q_{f,j}^{k}(S_{k},\;B_{k})=1-\;\prod_{i\in S_{k}}(1-P_{f,\left(i,j\right)}(\frac{\tau }{b_{k}}))$$

To represent the assignment policy, a three-dimensional matrix $A_{\mathrm{NXMXK}}\;$ is defined as

(12) $$A_{ijk}^{n}\{\begin{array}{c} 1\;if\;i\;\in S_{k\;\;\;}\;and\;j\;\in B_{k}\;\\ -\\ 0\;otherwise \end{array}$$

The problem is formulated and given as

(13) $$\max_{X_{s}X_{c},\;}=\sum_{k}R_{k}\;(S_{k}(X_{s}),B_{k}\left(X_{c})\right)$$

(14) $$\sum_{k=1}^{K}x_{s,i}^{k}=1,\;\forall \;i$$

(15) $$\sum_{k=1}^{K}x_{c,j}^{k}=1,\;\forall j$$

(16) $$\sum_{i\;\in S_{k}}x_{s,i}^{k\;}\geq \bar{m},\;\forall k$$

The optimization problem can be solved in polynomial time if and only if the corresponding decision problem can be solved. Thus, the proof of the algorithm for an optimization problem is equivalent to proving its corresponding decision problem.

## 4. Results and Discussion

### Simulation Parameters

The simulation results have been presented for the proposed cluster-based cooperative spectrum sensing with a greedy heuristic Algorithm 1 (GHA) based on the analytical expressions established in the previous section. The performance features such as the achievable throughput, average count of clusters and energy of the proposed cluster-based greedy heuristic algorithm have been appraised and linked with the conventional optimal algorithm using MATLAB. The assumptions made in the study are given in Table 1.
**Algorithm1**: Greedy algorithmInput:$G_{K}$ ($X_{k}\cup^{\;}Y_{k}$,$\in_{k}$),.$\;m_{k}$

Initialization: $S_{k}$ = $\;X_{k}$, $B_{k}$ = Ø, $A^{\left(k-1\right)}$, l ←1, $X_{s}^{k}$ = [1] and $X_{c}^{k}$= [0]$y_{l}$ = arg $max_{y\in Y_{k}}$ deg(y)

while |$S_{k}$| ≥ $\;m_{k}$ and |$Y_{k}$| > 0
$y_{l}$ = arg maxy∈YkBk
$ifdeg\left(y\right)m_{k}then$break;else$P_{k}=1;$$B_{k}\leftarrow B_{k}\cup^{\;}y_{l}$;$\;\;\;\;\;\;\Gamma_{k}$[l] = $R_{k}(S_{k}$,$B_{k}$);
$x_{c,y_{l}}^{k}$ = 1;
$x_{s,i}^{k}$ = 0; ∀ i $\in \psi_{y_{l}{}_{r}}$; update $\;\;A_{i\;j\;k}$;
end if
Find l*=arg $max_{l\;}\Gamma_{k}$[l];$S_{k}={\cap^{\;}}_{l=1}^{l*}\psi_{y_{l}{}_{r}}$;$B_{k}=\{CH_{\;P_{k}\left[l\right]}$, $CH_{\;P_{k}\left[2\right]}$ ……………………$\;CH_{\;P_{k}\left[l*\right]}$}.

return rules

**Figure 2:**
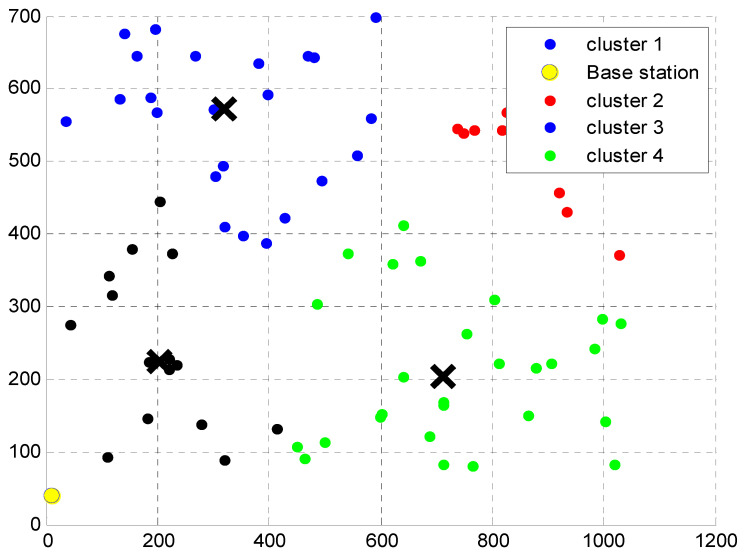
Simulation node setup vs. cluster formation.

In Figure 2, the node setup vs. cluster formation for the proposed CBCSS is plotted. The simulation area of 1200 m is used for the *X*-axis, and 700 m is used for the *Y*-axis for the proposed CBCSS.

**Figure 3:**
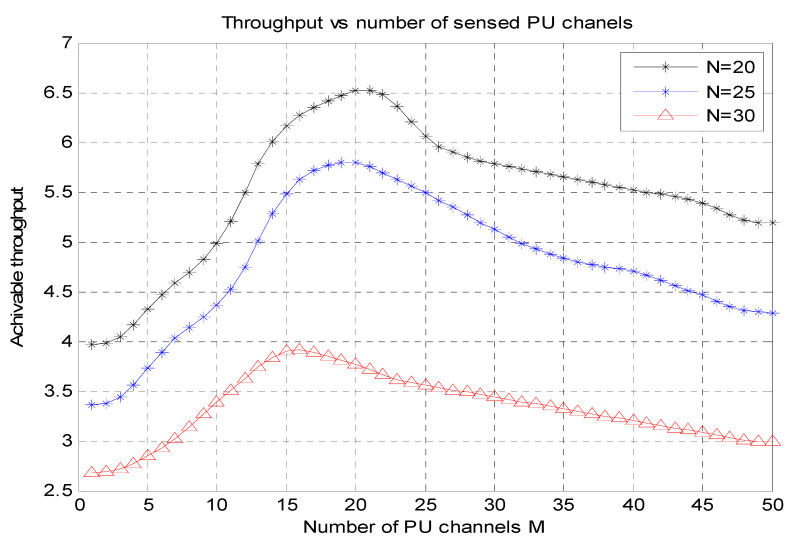
Number of sensed PU channels (M) vs. achievable throughput.

Figure 3 shows the total sensed PU channels (M) vs. the achievable throughput graphs for diverse values N = 20, N = 25, N = 30, where N represents the number of secondary channels. The number of sensed PU channels (M) is used for the *X*-axis, and the attainable throughput is used for the *Y*-axis.

**Figure 4:**
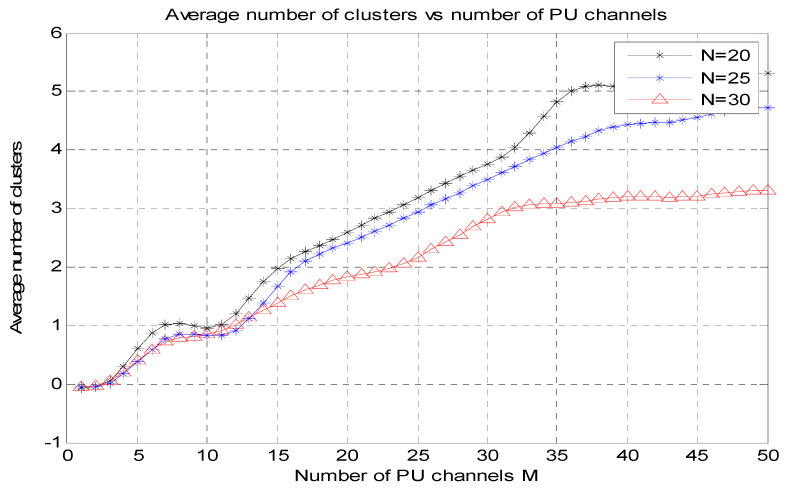
Number of PU channels (M) vs. average number of clusters.

Figure 4 shows the amount of PU channels (M) vs. the average figure of cluster graphs for N = 20, N = 25, N = 30. The number of PU channels (M) is used for the *X*-axis, and the average number of clusters is used for the *Y*-axis. 

It is apparent that when the number of PU channels rises, the average figure of clusters also rises because the number of clusters formed relies heavily on the detection range of the PU.

**Figure 5:**
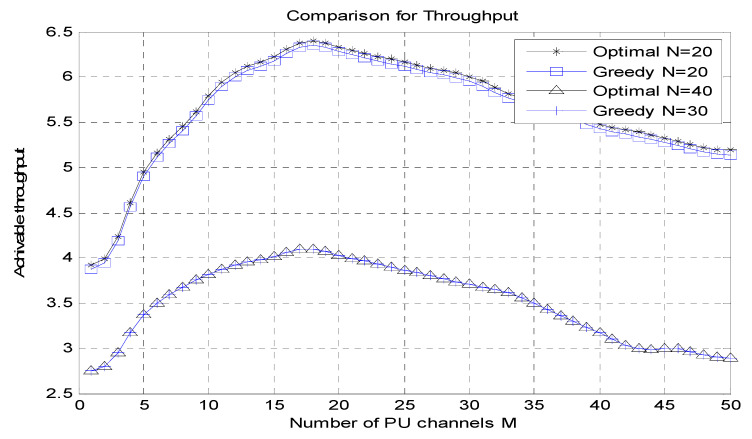
Number of PU channels (M) vs. achievable throughput.

Figure 5 shows the count of the PU channel (M) vs. achievable throughput graphs for optimal N = 20, greedy N = 20, optimal N = 40 and greedy N = 30 ranges. The number of PU channels (M) is used for the *X*-axis, and the achievable throughput is used correspondingly for the *Y*-axis. 

It has been perceived that the suggested algorithm attains an almost ideal performance, with an extreme performance loss of 4.6% for the achievable throughput.

**Figure 6:**
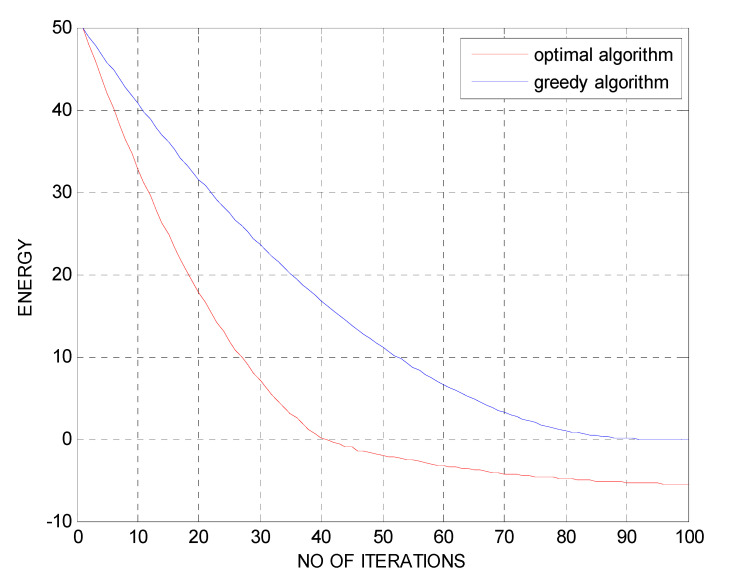
Number of iterations vs. energy (bits/J).

Figure 6 shows the number of iterations vs. energy (bits/J) graph for optimal and greedy algorithm techniques. The number of iterations is used for the *X*-axis, and the energy is used correspondingly for the *Y*-axis.

From the comparison graphs, it has been detected that the energy saved is expressed as a function of the number of iterations. Clearly, the number of iterations surges as the energy declines. The time necessary for sensing the PU channel is known as an iteration.

The results of the comparison between the proposed system and the existing systems are shown in Table 2. The simulation parameters, such as achievable throughput, the average number of clusters and energy, have been associated.

Table 2 reveals that the proposed CBCSS for 5G and beyond 5G IoT applications has high achievable throughput (96.87%), a high average number of clusters (5) and less energy (0.23 bits/J) than the existing schemes. Based on the simulation result, it is concluded that the proposed CBCSS algorithm provides a better solution for the SUs and is suitable for utilizing the PU channels effectively in 5G and beyond-5G IoT applications. Thus, the overall performance analysis suggests that the CBCSS algorithm for the cooperative spectrum sensing with a greedy heuristic algorithm for 5G and beyond-5G IoT applications in CRN performed well and achieved good performance in terms of achievable throughput, the average number of clusters and energy. Hence, for the effective 5G and beyond-5G IoT communication applications, the CBCSS scheme can enable additional benefits, such as the maximum achievable throughput.

## 5. Conclusions

In order to capitalize on the achievable throughput of cognitive radio networks, a CBCSS with GHA has been offered for 5G and beyond-5G IoT applications. The CBCSS algorithm was suitably developed. Performance parameters, such as achievable throughput, the average number of clusters and energy, have been scrutinized for the proposed CBCSS algorithm. In comparison with the ideal algorithm, the recommended CBCSS with GHA showed an achievable throughput of 96.87%. Thus, the proposed greedy algorithm performed better in terms of high achievable throughput and low energy.

## Figures and Tables

**Table 1:** Simulation parameters.

Description	Range
Simulation Area	1200 × 700 m
Primary Users (M)	50
Secondary Users (N)	40
Cluster Size	25 users/cluster
Number of Clusters	04
Transmission Range	250 m
Packet Size	165 bytes
Mobility Model	Random Waypoint
Node Pause Time	5 s
Sampling Frequency	6 MHz
Sensing Time	3–4 ms

**Table 2:** The comparison of the existing methods and proposed CBCSS.

Parameters	Song et al. [7]	Ruby et al. [12]	Li et al. [15]	Nadine et al. [17]	Proposed CBCSS
Achievable throughput	92.73%	84.83%	90.24%	82.90%	96.87%
Average number of clusters	2	1	3	3	5
Energy	1.87 bits/J	3.64 bits/J	0.94 bits/J	6.23 bits/J	0.23 bits/J
